# Impact of Mental and Physical Stress on Blood Pressure and Pulse Pressure under Normobaric versus Hypoxic Conditions

**DOI:** 10.1371/journal.pone.0089005

**Published:** 2014-05-09

**Authors:** Michael Trapp, Eva-Maria Trapp, Josef W. Egger, Wolfgang Domej, Giuseppe Schillaci, Alexander Avian, Peter M. Rohrer, Nina Hörlesberger, Dieter Magometschnigg, Mila Cervar-Zivkovic, Peter Komericki, Rosemarie Velik, Johannes Baulmann

**Affiliations:** 1 Research Unit of Behavioural Medicine, Health Psychology and Empirical Psychosomatics, Department of Medical Psychology and Psychotherapy, Medical University of Graz, Graz, Austria; 2 University Clinic of Psychiatry. Medical University of Graz, Graz, Austria; 3 Department of Pneumology, Medical University of Graz, Graz, Austria; 4 Department of Medicine, University of Perugia and Terni University Hospital, Terni, Italy; 5 Institute for Medical Informatics, Statistics and Documentation, Medical University of Graz, Graz, Austria; 6 Institute for Hypertension, Vienna, Austria; 7 Department of Obstetrics and Gynecology, Medical University of Graz, Graz, Austria; 8 Department of Dermatology, Medical University of Graz, Graz, Austria; 9 CTR Carinthian Tech Research, Villach/St. Magdalen, Austria; 10 UKSH Universitätsklinikum Schleswig-Holstein, Campus Lübeck, Lübeck, Germany; University of Southampton, United Kingdom

## Abstract

**Objective:**

Hypobaric hypoxia, physical and psychosocial stress may influence key cardiovascular parameters including blood pressure (BP) and pulse pressure (PP). We investigated the effects of mild hypobaric hypoxia exposure on BP and PP reactivity to mental and physical stress and to passive elevation by cable car.

**Methods:**

36 healthy volunteers participated in a defined test procedure consisting of a period of rest 1, mental stress task (KLT-R), period of rest 2, combined mental (KLT-R) and physical task (bicycle ergometry) and a last period of rest both at Graz, Austria (353 m asl) and at the top station Dachstein (2700 m asl). Beat-to-beat heart rate and BP were analysed both during the test procedures at Graz and at Dachstein and during passive 1000 m elevation by cable car (from 1702 m to 2700 m).

**Results:**

A significant interaction of kind of stress (mental vs. combined mental and physical) and study location (Graz vs. Dachstein) was found in the systolic BP (p = .007) and PP (p = .002) changes indicating that during the combined mental and physical stress task sBP was significantly higher under hypoxic conditions whereas sBP and PP were similar during mental stress both under normobaric normoxia (Graz) and under hypobaric hypoxia (Dachstein). During the passive ascent in cable car less trivialization (psychological coping strategy) was associated with an increase in PP (p = .004).

**Conclusion:**

Our data show that combined mental and physical stress causes a significant higher raise in sBP and PP under hypoxic conditions whereas isolated mental stress did not affect sBP and PP under hypoxic conditions. PP-reaction to ascent in healthy subjects is not uniform. BP reactions to ascent that represents an accumulation of physical (mild hypobaric hypoxia) and psychological stressors depend on predetermined psychological traits (stress coping strategies). Thus divergent cardiovascular reactions can be explained by applying the multidimensional aspects of the biopsychosocial concept.

## Introduction

Hypertension is the most important modifiable risk factor for cardiovascular morbidity and mortality worldwide [1]. The pulsatile component of arterial blood pressure (BP), i.e. pulse pressure (PP), has been recognized as an important predictor of future cardiovascular events [2]–[4], and its predictive power is stronger than that of diastolic blood pressure (dBP) [5]. PP is a complex parameter which is determined by ventricular ejection and by arterial stiffness/pulse wave reflection [6], and is associated with aging and increased large-artery pulse wave velocity (PWV) [7], [8].

Hypoxia induces the release of vasoactive substances that are described to induce local vasodilatation to ensure oxygen delivery to different tissues [9], [10]. Furthermore hypoxia induces the activation of the sympathetic nervous system (SNS) resulting in increased BP and heart rate (HR) [11] and in an increased sympathetic induced vasoconstrinctive effect towards skeletal muscle [9], [12]. Recent studies have shown that acute hypobaric hypoxia provokes an increase in resting HR and BP during real altitude exposure or simulated altitude exposure in a hypobaric chamber [13].

The common view on cardiovascular responses to high altitude includes increases in cardiac output (CO), HR and BP [14] caused by both acute and chronic hypoxia [15]. Hence, a fast ascent by cable car may be associated with increased HR and decreased heart rate variability (HRV) [16].

According to the extended biopsychosocial model which assumes the unity of mind and body [17], [18] every psychophysiological event includes psychological and physiological reactions at the same time. Acute psychosocial stress exposure is associated with an activation of the SNS leading to different physiological effects, like, for instance, a rise in HR and BP, pro-arrhythmogenic potential, vasoconstriction and haemostatic changes - chronic stress and affective disorders are linked to a higher cardiovascular reactivity to mental stressors [19], [20]. The reactions to external challenges e.g. change in atmospheric pressure, temperature and/or oxygen concentration, also include perceptive processes with their cognitive-emotional sequels, individual coping processes and subjective experiences. Thus a high emotion-orientated coping score is associated with an increased risk of hypertension [21], whereas high levels of self-efficacy in problem-focused coping are associated with a decrease in mean arterial pressure, systolic blood pressure (sBP), and PP [22].

The aim of our study was to determine cardiovascular responses to a mental and a combined mental and physical stress task both under normobaric normoxia (Graz 383 m above sea level [asl] and mild hypobaric hypoxia in an alpine setting (Dachstein 2,700 m asl). Furthermore we searched for potential, underlying, psychological-stress coping parameters influencing PP alterations during passive ascent.

## Methods

### Study population

From a total of 41 participants 36 healthy subjects (22 men, 14 women) were included in the analysis. All subjects fulfilled the following inclusion criteria: no clinical signs of immediately preceding infectious diseases, age 18–50 years, adequate compliance and understanding of the German language, body mass index (BMI)<30 kg/m^2^, and absence of acute psychiatric disorders, seizure disorders, unstable asthma and chronic obstructive pulmonary disease. The following five subjects were excluded from analysis: two men did not complete the psychological questionnaires (SVF and FPI), one woman refused to enter the cable car because of situational anxiety, one woman showed an anxious reaction (type fight and flight) during the ascent in the cable car and one man suffered an anxious reaction (type loss of control) at the mountain top station. Details on biometric parameters are given in table 1.

**Table 1:** Biometric data of the participants.

	mean	SD	min	max
age (years)	26.7	3.9	19.0	38.3
height (cm)	175.1	8.4	158.0	191.0
weight (kg)	69.9	12.1	47.0	95.0
body surface area (m^2^)	1.9	0.2	1.5	2.2

### Ethics statement

In a presentation, subjects were informed in detail about the study, both orally and by written informed consent. The study was approved by the local ethics committee (“Ethikkommission der Medizinischen Universität Graz” EK 19–218 ex 07/08).

### Physiological methods and test procedure

**Figure 1:**
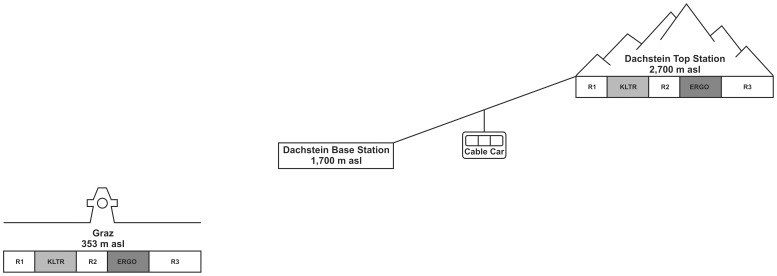
Test design – Graz, cable car and Dachstein.

Volunteers participated in a defined test procedure both at Graz, Austria (353 m asl) and at the summit station Dachstein – Hunerkogel, Austria (2,700 m asl). The study was conceptualized as a cross-over design (Group1: Graz – Dachstein, Group2: Dachstein – Graz). The test procedure is graphically shown in figure 1.

### Test procedure

After the collection of socio-demographic data, the electrodes (ecg, impedance cardiography) and the sensors (for oscillometric [upper part of the dominant arm], continual plethysmographic [finger cuffs placed on the non-dominant hand] blood pressure measurement, skin conductance and skin temperature [data not shown]) were put in place. During the test procedure cardiovascular parameters were continuously recorded with the Task Force Monitor, CNSystems Medizintechnik AG, Graz, Austria. Signal quality was permanently controlled during the procedure. If it was necessary, sensors were adjusted before or after the relevant periods in case of inefficient data quality in order to avoid biasing the data.

The following periods had to be completed (see figure 1):

1. period of rest (6 minutes) in a sitting position with eyes closed (R1)
2. mental stress task: KLT-R (first four coulombs, 8 minutes)
3. period of rest (6 minutes) in a sitting position with eyes closed (R2)
4. combined mental (KLT-R, last four coulombs) and physical task (ERGO) (warming up: 3 minutes, combined task: 8 minutes)
5. period of rest (10 minutes) in a sitting position with eyes closed (R3)

### Physical task (bicycle ergometer)

#### Warming up

3 minutes increasing intensity (1^st^ minute at 0.75 W/kg, 2^nd^ minute at 1 W/kg, 3^rd^ minute Watt modulation to reach the individual target heart rate).

#### Physical strain

Heart rate was constantly kept at 65% of the corresponding age-related maximum heart rate (according to the recommendations of “Arbeitsgemeinschaft für Ergometrie der Österreichischen Kardiologischen Gesellschaft, Koordinator M. Niederberger, Kardiolog. Univ.-Klinik Wien”) for 8 minutes.

Before KLT-R and after ERGO a pulse oximetry measurement (BCI Digit 3420 Finger Pulse Oximeter) was performed in order to estimate the participants' hemoglobin saturation.

### Data recording during passive ascent in cable car

After the arrival at the cable car base station (Türlwandhütte, Dachstein, Ramsau, Austria; 1,702 m asl) ECG-electrodes of a mobile ECG-recorder (TOM*medical*, Graz, Austria) and a beat-to-beat BP measuring device – a CNAP system (CNAP Monitor 500, CNSystems Medizintechnik AG, Graz, Austria) that consists of a double finger cuff, a pressure transducer mounted on the forearm and a non-invasive BP cuff for calibration [23] – were positioned in a special laboratory, specifically prepared for the study, with a comfortable room temperature of 20–25°Celsius. To avoid occurrences of acoustic (noises of other passengers) and optical distractions during the ascent, ear protectors and sun glasses were worn by the subjects. In order to avoid orthostatic influences during entrance into the cable car, the participants remained seated in a wheel chair, in which they had been sat since arrival at the laboratory and before the application of the sensors. Remaining in this standardized comfortable sitting position, participants entered the cable car (capacity: approx. 70 passengers). The ascent was observed by four investigators who controlled the test procedure and who guaranteed the avoidance of interactions between other passengers and the participants. From base station Türlwandhütte (1,702 m) to top station Hunerkogel (2,700 m) participants ascended a difference in altitude of approximately 1,000 m (horizontal length: 1,933.76 m; diagonal length/route: 2,174.91 m; average gradient: 51.5%) with a speed of 6–10 m/s (duration of ascent approximately 7 minutes) [24], [25]. ECG and BP were recorded continuously during the ascent, and HR was obtained from the ECG signal. The vertical position of the fingers as compared with the heart was kept constant during ascent, in order to avoid gravitational influences.

### Psychometric methods

#### Mental Stress Test – Konzentrationsleitungstest (KLT-R) [26]

The “Konzentrations-Leistungs-Test - Revidierte Fassung” (KLT-R) is a paper-pencil concentration-performance-test which deals with both quantity and quality of long-term strain and the power of concentration. The KLT-R consists of nine blocks containing 20 separate items (arithmetic tasks). Depending on the intermediate result of each calculation, further calculation rules have to be applied. The participants have to proceed to the next block exactly after two minutes, regardless of their progress. Thus the participants have to coordinate several single operations simultaneously. In our study, the signals to start, proceed and finish the test were provided by an mp3-file to guarantee precise intervals. The participants were provided different but parallel versions of the KLT-R at both Dachstein and in Graz in order to avoid any learning effects they might have developed.

#### German stress-coping questionnaire SVF 120

The psychometric tests that measure psychological traits were performed in Graz immediately after the test procedure. The basis of the stress coping concept of the current study was the transactional model of stress by Lazarus and Folkman [27]. Concerning stress coping strategies we can differ between positive/adaptive strategies that result in efficient stress reduction and negative/maladaptive strategies that are described to be associated with a stress-enhancing behaviour [28]. Coping strategies were assessed using the German stress-coping questionnaire SVF 120 (“Stressverarbeitungsfragebogen 120”). The SVF 120 comprises 120 items and is divided into 20 subtests. Each item offers the possibility to select the adequate answer within a five-level rating scale [28].

#### Freiburger Personality Inventory – Revised Version (FPI-R)

In order to evaluate personality traits the German version of the Freiburger Personality Inventory (FPI) was used [29]. The test comprises 138 items. The standardscales of the FPI-R (revised) are: life satisfaction, social orientation, achievement orientation, inhibitedness, excitability, aggressiveness, strain, somatic complaints, health concern, frankness. The two subscales are extraversion and emotionality [30].

All applied instruments represent fully standardized psychometric methods that are widely utilized and validated.

#### Data processing

During the test procedure at Graz and at Dachstein, ECG and BP were recorded continuously. In order to guarantee optimal data quality we calculated the mean values and standard deviation (SD) of HR, sBP, dBP and PP by using the last five minutes of R1, R2 and R3.

Data analysis of the physiological variables taken during the cable car ascent was performed in MATLAB. Both sBP and dBP of each heart beat were calculated from the filtered BP raw data by applying an algorithm determining the global minimum and maximum of the BP signal for each heart beat: 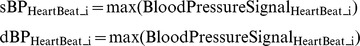



After calculating the data for every heart beat during the ascent by cable car, the whole measurement period was divided into three equal time intervals (TI1–TI3). By dividing into TI1, TI2 and TI3, minimal differences in the ascent time of the cable car can be brought to light and comparable time segments can be obtained. The mean value and standard deviation (SD) of sBP and dBP were determined for each interval by using the predefined MATLAB functions *mean*() and *std*().

### Statistical analysis

The data is presented as mean and SD for continuous data and as a frequency for categorical data. Changes from pre stress to stress were calculated for HR, dBP, sBP and PP. To analyse differences between the study locations, the kind of stress and the interaction of study location*kind of stress, a two factor repeated measurement ANOVA was used.

To analyse the response to cable car ascent in HR, sBP, dBP and PP, the difference of the 1^st^ and the 3^rd^ TI was calculated. To characterize subjects with an increase or decrease in these variables, subjects were grouped into an “increasing” or “decreasing” group (cutpoint location: 0). A model relating the subject's changes in PP (increase/decrease) to independent psychological predictors was built using logistic regression. In the first step univariate logistic regression analyses were performed. The impact of each coping style and personality aspect on the direction of change in PP was analysed. Significant variables or variables which showed a specific tendency were selected for multivariate logistic regression. Variables in the final model were selected with a backward stepwise procedure. The decision to remove variables was based on a likelihood-ratio test. A p-value of less than 5% was considered significant. For data analysis SPSS 20 (SPSS Inc, Chicago, IL) was used.

## Results

### Analysis of test procedures

All analysed variables showed a more pronounced change during combined mental and physical stress regardless of location: HR (p<.001), dPB (p = .018), sBP (p<.001) and PP (p<.001) increased (table 2). Concerning the different locations (Graz vs. Dachstein) no significant changes in dBP (p = .885), sBP (p = .159) and PP (p = .097) were found.

**Table 2:** Analysis of differences between study locations, kind of stress and the interaction of study location*kind of stress (two factor repeated measurement ANOVA).

study location (a)	Graz		Dachstein		p-value		
kind of stress (b)	mental stress	combined mental and physical stress	mental stress	combined mental and physical stress	a	b	a*b
	mean (SD)	mean (SD)	mean (SD)	mean (SD)			
diastolic blood pressure (mmHg)	7.6 (8.3)	14.3 (16.4)	7.1 (19.9)	15.6 (21.0)	.885	.018	.773
systolic blood pressure (mmHg)	11.7 (11.9)	29.9 (21.9)	7.0 (18.6)	42.4 (19.3)	.159	<.001	.007
pulse pressure (mmHg)	4.0 (5.9)	15.6 (16.6)	−0.0 (13.3)	26.8 (16.2)	.097	<.001	.002
heart rate	8.0 (9.0)	53.0 (7.1)	9.5 (7.3)	53.2 (8.5)	.639	<.001	.619

A significant interaction between kind of stress (mental vs. combined mental and physical) and study location was found in the changes of sBP (p = .007) and PP (p = .002). While the sBP and PP reaction to mental stress was slightly higher in Graz as compared to Dachstein, the sBP and PP reaction to combined mental and physical stress was significantly lower in Graz as compared to Dachstein. Details on differences of biometrical data (HR, sBP, dBP and BRS) measured during the first period of rest R1 are given in table 3.

**Table 3:** Differences of biometrical data (HR, sBP, dBP and BRS) measured during the first period of rest R1.

		mean	SD	minimum	maximum	p-value
heart rate	Graz	70.0	8.9	55.0	91.9	.090
	Dachstein	67.0	9.4	47.2	81.3	
diastolic blood pressure	Graz	73.8	10.1	54.9	93.2	.228
	Dachstein	77.1	16.3	50.2	149.9	
systolic blood pressure	Graz	112.8	14.2	87.9	165.7	.181
	Dachstein	116.9	14.0	96.8	151.5	
pulse pressure	Graz	39.0	8.1	25.0	72.4	.788
	Dachstein	39.8	13.3	1.6	83.5	

Pulse oximetry showed higher values in Graz (p<.001) than at Dachstein and was also higher before mental stress than after combined mental and physical stress (p<.001). This reduction was more pronounced at Dachstein as compared to Graz (p = .009). Details on absolute values of pulse oximetry are given in table 4.

**Table 4:** Absolute values of pulse oximetry (Graz vs. Dachstein).

		mean	SD
Graz	before mental stress	96.6	1.1
	after combined mental and physical task	96.1	1.6
Dachstein	before mental stress	93.2	1.9
	after combined mental and physical task	90.9	3.1

### PP-reactivity during ascent in cable car, stress coping strategies and personality

There were no significant changes *on average* in HR, sBP, dBP and PP. According to univariate analysis, *less trivialization* was associated with an increase in PP during ascent (p = .004). Similar, although non-significant associations were found between PP increase and *social withdrawal* (p = .059), *relaxation* (p = .060), and *intrusive thoughts* (p = .098). There was no effect due to personality. Multivariate logistic regression analysis revealed that 70.3% of the variance in changes in PP could be explained – using a logistic regression model – by *trivialization* (p = .003) and *social withdrawal* (p = .018). Details for the multivariate logistic regressions are given in table 5.

**Table 5:** Multivariate logistic regression for changes in pulse pressure during ascent in cable car.

	OR	95% CI	p
trivialization	.420	.239–.738	.003
social withdrawal	1.543	1.077–2.211	.018

## Discussion

Our results show that during combined mental and physical stress sBP and PP were higher under hypoxic conditions compared to normobaric normoxia, whereas sBP and PP were similar during mental stress both under normobaric normoxia (Graz) and under hypobaric hypoxia (Dachstein). Thus we can assume that the sBP and PP reaction to mental stress are comparable under these different conditions. In contrast combined mental and physical stress has a stronger influence on sBP and PP under moderate hypoxic conditions. The proportion of stress that is induced by hypobaric hypoxia in the present study can be considered to be relatively small as it is generally accepted that up to about 2,500 m, there are few, if any, effects of hypoxia [11], [13]. Above 3,000 m only some effects of hypoxia are likely to be experienced by most unacclimatised visitors [11], [13].

Furthermore it was shown that the PP-reaction to ascent was not uniform. Both an increase and decrease in PP can occur during the procedure. PP reactions to ascent depended on predetermined psychological traits. In the present study, we identified specific stress coping strategies to determine the different PP reactions to ascent. In detail, our data show that high values of trivialization and relaxation induced, with a high probability, a PP reduction during the ascent by cable car. The (psychological) tendency to avoid social interaction and to show intrusive thoughts in stressful situations was associated with experiencing the situation in the cable car as stressful, which again can be speculated to be associated with an increase in emotional and vegetative arousal and hence with an increase in PP.

Several studies have shown distinct psychological influences on BP regulation and some studies investigated associations between psychological traits and arterial stiffness and pulse wave reflection [31]–[35], although those studies were not performed in an alpine setting. An acute mental stress can cause an increase in aortic stiffness and pulse wave reflection leading to changes in PP, associated with increased cardiovascular risk [19], [20], [34]. If the observed changes in PP are mediated by the vasculature and thus probably even stronger pronounced in central pressure needs to be addressed in further studies.

Some psycho-cardiological interventional studies do exist, which again were performed in normobaric conditions. Blumenthal et al. found that a combined intervention of physical and stress management training improved cardiovascular risk markers in a more efficient way than the standard medical care [36].

BP reactions depend on coping behavior [37], and stress reactivity of hemodynamic parameters is determined by structural and functional aspects of arterial vessels and baroreflex sensitivity (BRS) [38]. Tasks demanding active coping strategies increase cardiac output and decrease vascular resistance resulting in an increase in BP [37].

A high degree of pessimism is associated with a higher ambulatory BP [39]. Low self-efficacy and high incentive value is associated with a stronger increase in PP in stressful tasks and high cardiovascular reactivity, affecting PP and sBP but not dBP [40], [41]. Otherwise, a high level of self-efficacy for problem-focused coping is associated with a low mean arterial pressure, sBP and PP [22]. Social support is associated with a low PP reactivity to a mental stress (reading) task in people with high levels of loneliness [42].

Our results add to the body of literature the conclusion that psychological patterns keep influencing or regulating BP changes with an ascent that is accompanied by light hypobaric hypoxia. We could moreover show that not just the degree of BP-change but also the direction of BP-changes are mainly regulated by preexisting psychological traits. Overstimulated allostatic systems can lead to an allostatic load [43]. Both resilience and risk factors play an important role in the autoregulative process of sustaining and/or inducing health [17], [18]. Thus the effects of stress (individual strain) seem to depend on both objective conditions/stressors (cable car permanence, hypobaric hypoxia) and on psychological variables (stress coping strategies), that determine the direction and reactivity of psycho-vegetative and psycho-cardiologic parameters. PP reactivity during ascent in a cable car did not depend only on physical parameters, as for instance a light hypobaric hypoxia, but also on factors such as coping strategies (for example trivialization and social isolation). In general, the cable car permanence could be viewed as a psychological pattern disruption. In comparable situations different stress factors accumulate. PP reactivity (respectively arterial stiffness and wave reflection) appears within the situation – limited in time – to be influenced by long standing psychological variables (traits), what again should be addressed in future studies to explore the underlying physiological regulation.

It was shown that mild hypobaric hypoxia did not affect BP significantly at 2,700 m asl during a mental stress task. In contrast, a combined mental and physical stress situation seems to influence the BP under light hypoxic conditions. Physical strain (at constant HR, both under normobaric and hypobaric conditions) causes a significant increase in sBP and PP at 2,700 m asl. Thus we can hypothesize that in healthy subjects an objective cognitive stressor can be well tolerated and does not affect BP significantly, whereas a physical stressor causes a higher BP only under mild hypoxia. The PP reaction during passive ascent in a cable car (a change of 1,000 m in altitude) seems to be influenced more intensively by psychological factors than by changes in hypoxia.

Limitations: Due to the fact that ventilation was not measured during this study we cannot estimate an eventual influence of ventilation on cardiovascular parameters and oxygen saturation. These aspects must be investigated in future studies. Because mental stress has a prolonged unfavourable effect on arterial stiffness and wave reflection, and hence on BP and PP, we cannot fully exclude a carry-over effect. Present data are given for the maximum of 1 hour following mental stress or 2 hours [34], [44]. In the present study a carry-over effect seems to be unlikely, because the median time between our test sequences was 12.5 days besides using a cross-over study design. Due to the relatively small number of subjects used in running a multivariate analysis and the exploratory character of the study, further studies may be needed to replicate these findings.

Conclusion: Analyses of biopsychological interdependent relations between environmental stress, psychological behavior and cardiovascular reactions lead to very new perspectives on cardiovascular effects. This study shows that a uniform stress causes divergent cardiovascular reactions depending on psychological dimensions and the kind and intensity of the stressor (mental vs. physical). Thus the divergent cardiovascular reactions can be explained by applying the multidimensional aspects of the biopsychosocial concept and further investigations in mountain medicine and BP research should take into account biological, psychological and socio-economic aspects in order to explain the complex BP-reactivity [45].

## References

[1] Lopez AD, Mathers CD, Ezzati M, Jamison DT, Murray CJ (2006) Global and regional burden of disease and risk factors, 2001: systematic analysis of population health data. Lancet 367: 1747–1757. 16731270 10.1016/S0140-6736(06)68770-9

[2] Skoglund PH, Ostergren J, Svensson P (2012) Ambulatory pulse pressure predicts cardiovascular events in patients with peripheral arterial disease. Blood Press. 10.3109/00365599.2012.676755 22553945

[3] Vlachopoulos C, Aznaouridis K, O'Rourke MF, Safar ME, Baou K, et al (2010) Prediction of cardiovascular events and all-cause mortality with central haemodynamics: a systematic review and meta-analysis. Eur Heart J 31: 1865–1871. 20197424 10.1093/eurheartj/ehq024

[4] Verdecchia P, Angeli F (2007) Does brachial pulse pressure predict coronary events? Adv Cardiol 44: 150–159. 17075205 10.1159/000096727

[5] Little MO (2011) Hypertension: how does management change with aging? The Medical clinics of North America 95: : 525–537, x. 10.1016/j.mcna.2011.02.002 21549876

[6] Van Bortel LM, Struijker-Boudier HA, Safar ME (2001) Pulse pressure, arterial stiffness, and drug treatment of hypertension. Hypertension 38: 914–921. 11641309 10.1161/hy1001.095773

[7] Lee HY, Oh BH (2010) Aging and arterial stiffness. Circulation journal: official journal of the Japanese Circulation Society 74: 2257–2262. 20962429 10.1253/circj.cj-10-0910

[8] Raij L, Gonzalez-Ochoa AM (2011) Vascular compliance in blood pressure. Current opinion in nephrology and hypertension 20: 457–464. 21738031 10.1097/MNH.0b013e3283499d7b

[9] Casey DP, Joyner MJ (2012) Compensatory vasodilatation during hypoxic exercise: mechanisms responsible for matching oxygen supply to demand. J Physiol 590: 6321–6326. 22988134 10.1113/jphysiol.2012.242396 PMC3533194

[10] Crawford JH, Isbell TS, Huang Z, Shiva S, Chacko BK, et al (2006) Hypoxia, red blood cells, and nitrite regulate NO-dependent hypoxic vasodilation. Blood 107: 566–574. 16195332 10.1182/blood-2005-07-2668 PMC1895612

[11] Hainsworth R, Drinkhill MJ (2007) Cardiovascular adjustments for life at high altitude. Respiratory physiology & neurobiology 158: 204–211. 17597013 10.1016/j.resp.2007.05.006

[12] Hanada A, Sander M, Gonzalez-Alonso J (2003) Human skeletal muscle sympathetic nerve activity, heart rate and limb haemodynamics with reduced blood oxygenation and exercise. J Physiol 551: 635–647. 12909683 10.1113/jphysiol.2003.044024 PMC2343217

[13] Hainsworth R, Drinkhill MJ, Rivera-Chira M (2007) The autonomic nervous system at high altitude. Clinical autonomic research: official journal of the Clinical Autonomic Research Society 17: 13–19. 17264976 10.1007/s10286-006-0395-7 PMC1797062

[14] Naeije R (2010) Physiological adaptation of the cardiovascular system to high altitude. Progress in cardiovascular diseases 52: 456–466. 20417339 10.1016/j.pcad.2010.03.004

[15] Calbet JA (2003) Chronic hypoxia increases blood pressure and noradrenaline spillover in healthy humans. The Journal of physiology 551: 379–386. 12844510 10.1113/jphysiol.2003.045112 PMC2343162

[16] Guger C, Domej W, Lindner G, Pfurtscheller K, Pfurtscheller G, et al (2005) Effects of a fast cable car ascent to an altitude of 2700 meters on EEG and ECG. Neuroscience letters 377: 53–58. 15722187 10.1016/j.neulet.2004.11.065

[17] Egger JW (2005) Das biopsychosoziale Krankheitsmodell – Grundzüge eines wissenschaftlich begründeten ganzheitlichen Verständnisses von Krankheit. Psychologische Medizin 16: 3–12.

[18] Egger JW (2008) Grundlagen der “Psychosomatik” - Zur Anwendung des biopsychosozialen Krankheitsmodells in der Praxis. Psychologische Medizin 19: 12–22.

[19] Rozanski A, Kubzansky LD (2005) Psychologic functioning and physical health: a paradigm of flexibility. Psychosom Med 67 Suppl 1 S47–53. 15953801 10.1097/01.psy.0000164253.69550.49

[20] Rozanski A, Kubzansky LD (2005) Psychologic functioning and physical health: a paradigm of flexibility. Psychosomatic medicine 67 Suppl 1 S47–53. 15953801 10.1097/01.psy.0000164253.69550.49

[21] Ariff F, Suthahar A, Ramli M (2011) Coping styles and lifestyle factors among hypertensive and non-hypertensive subjects. Singapore medical journal 52: 29–34. 21298238

[22] Harmell AL, Mausbach BT, Roepke SK, Moore RC, von Kanel R, et al (2011) The relationship between self-efficacy and resting blood pressure in spousal Alzheimer's caregivers. British journal of health psychology 16: 317–328. 21489059 10.1348/135910710X504932 PMC3236810

[23] Jeleazcov C, Krajinovic L, Munster T, Birkholz T, Fried R, et al (2010) Precision and accuracy of a new device (CNAPTM) for continuous non-invasive arterial pressure monitoring: assessment during general anaesthesia. British journal of anaesthesia 105: 264–272. 20627878 10.1093/bja/aeq143

[24] Planai-Hochwurzen-Bahnen Gesellschaft m.b H (2009). Schladming, Austria: BearingPoint INFONOVA GmbH.

[25] Planai-Hochwurzen-Bahnen Gmb H Operating instructions for Dachstein-Südwandbahn - Approved by decision of the Federal Ministry for Transport, Innovation and Technology.

[26] Düker H, Lienert GA (2001) KLT-R Konzentrations-Leistungs-Test - Revidierte Fassung. Göttingen, Bern, Toronto, Seattle: Hogrefe - Verlag für Psychologie.

[27] Lazarus RS, Folkman S (1984) Stress, appraisal, and coping. New York Springer Publishing Company.

[28] Erdmann G, Janke W (2008) SVF - Stressverarbeitungsfragebogen - Stress, Stressverarbeitung und ihre Erfassung durch ein mehrdimensionales Testsystem. Göttingen: Hogrefe Verlag GmbH & Co.KG.

[29] Fahrenberg J, Hampel R, Selg H (2001) Das Freiburger Persönlichkeitsinventar. Göttingen: Hogrefe. 148 p.

[30] Fahrenberg J, Hampel R, Selg H (2010) FPI-R Freiburger Persönlichkeitsinventar: Hogreve Verlag GmbH & Co. KG.

[31] Midei AJ, Matthews KA (2009) Social relationships and negative emotional traits are associated with central adiposity and arterial stiffness in healthy adolescents. Health Psychol 28: 347–353. 19450041 10.1037/a0014214 PMC2818581

[32] Dietz LJ, Matthews KA (2011) Depressive symptoms and subclinical markers of cardiovascular disease in adolescents. J Adolesc Health 48: 579–584. 21575817 10.1016/j.jadohealth.2010.09.001 PMC3096828

[33] Lewis TT, Sutton-Tyrrell K, Penninx BW, Vogelzangs N, Harris TB, et al (2010) Race, psychosocial factors, and aortic pulse wave velocity: the Health, Aging, and Body Composition Study. J Gerontol A Biol Sci Med Sci 65: 1079–1085. 20522528 10.1093/gerona/glq089 PMC2950813

[34] Vlachopoulos C, Kosmopoulou F, Alexopoulos N, Ioakeimidis N, Siasos G, et al (2006) Acute mental stress has a prolonged unfavorable effect on arterial stiffness and wave reflections. Psychosomatic medicine 68: 231–237. 16554388 10.1097/01.psy.0000203171.33348.72

[35] Vlachopoulos C, Xaplanteris P, Alexopoulos N, Aznaouridis K, Vasiliadou C, et al (2009) Divergent effects of laughter and mental stress on arterial stiffness and central hemodynamics. Psychosomatic medicine 71: 446–453. 19251872 10.1097/PSY.0b013e318198dcd4

[36] Blumenthal JA, Sherwood A, Babyak MA, Watkins LL, Waugh R, et al (2005) Effects of exercise and stress management training on markers of cardiovascular risk in patients with ischemic heart disease: a randomized controlled trial. JAMA: the journal of the American Medical Association 293: 1626–1634. 15811982 10.1001/jama.293.13.1626

[37] Sherwood A, Dolan CA, Light KC (1990) Hemodynamics of blood pressure responses during active and passive coping. Psychophysiology 27: 656–668. 2100351 10.1111/j.1469-8986.1990.tb03189.x

[38] Lipman RD, Grossman P, Bridges SE, Hamner JW, Taylor JA (2002) Mental stress response, arterial stiffness, and baroreflex sensitivity in healthy aging. The journals of gerontologySeries A, Biological sciences and medical sciences 57: B279–284. 10.1093/gerona/57.7.b279 12084798

[39] Raikkonen K, Matthews KA (2008) Do dispositional pessimism and optimism predict ambulatory blood pressure during school days and nights in adolescents? Journal of personality 76: 605–630. 18399951 10.1111/j.1467-6494.2008.00498.x PMC2919826

[40] Bandura A (1977) Self-efficacy: toward a unifying theory of behavioral change. Psychological review 84: 191–215. 847061 10.1037//0033-295x.84.2.191

[41] Sanz A, Villamarin F, Alvarez M (2006) Effects of specific and non-specific perceived control on blood pressure response in a stressful mental task. Biological psychology 71: 20–28. 16360877 10.1016/j.biopsycho.2005.01.010

[42] O'Donovan A, Hughes B (2007) Social support and loneliness in college students: effects on pulse pressure reactivity to acute stress. International Journal of Adolescent Medicine and Health 19: 523–528. 18348427 10.1515/ijamh.2007.19.4.523

[43] McEwen BS (1998) Stress, adaptation, and disease. Allostasis and allostatic load. Ann N Y Acad Sci 840: 33–44. 9629234 10.1111/j.1749-6632.1998.tb09546.x

[44] Reppel M, Franzen K, Bode F, Weil J, Kurowski V, et al (2013) Central hemodynamics and arterial stiffness during the finals of the world cup soccer championship 2010. Int J Cardiol 166: 627–632. 22196396 10.1016/j.ijcard.2011.11.096

[45] International Society of Biopsychosocial M (2011) Venice Declaration.

